# Primary CNS Burkitt Lymphoma Presenting as Sudden Bilateral Blindness in a Patient With Underlying Kabuki Syndrome: A Case Report

**DOI:** 10.7759/cureus.63725

**Published:** 2024-07-03

**Authors:** Abdulmalik H Alghamdi, Mazen Alzahrani, Yumna F Kamal, Reham Aljehani, Islam A Ibrahim, Abdullah S Alqahtani

**Affiliations:** 1 Ophthalmology, College of Medicine, Taif University, Taif, SAU; 2 Ophthalmology, Jeddah Eye Hospital, Jeddah, SAU; 3 Ophthalmology, King Abdulaziz Medical City, Ministry of National Guard - Health Affairs, Jeddah, SAU; 4 Faculty of Medicine, King Abdulaziz University Hospital, Jeddah, SAU; 5 Adult Cardiology, King Fahad Armed Forces Hospital, Jeddah, SAU; 6 Ophthalmology, King Abdullah International Medical Research Center, Jeddah, SAU; 7 Vitreoretinal and Ocular Oncology Surgery, King Saud Bin Abdulaziz University for Health Sciences, Jeddah, SAU

**Keywords:** blindness, primary central nervous system lymphoma, cancer predisposition, burkitt lymphoma, kabuki syndrome

## Abstract

Burkitt lymphoma is an aggressive B-cell non-Hodgkin lymphoma (NHL). Primary CNS lymphoma (PCNSL) is a rare disease, and the subtype of Burkitt lymphoma presenting as a sole CNS lesion is an even rarer diagnosis. Acute sudden blindness is a rare presenting symptom of PCNSL or NHL in general. We present an interesting case of a four-year-old boy with dysmorphic features whose visual examination showed a sudden bilateral loss of vision. There was bilateral eye proptosis and complete ptosis. Extraocular muscles were fixed straight. The pupils were fixed and mid dilated bilaterally and there was grade 3/4 papilledema in both eyes. Neuroimaging showed a mass in the base of the skull, extending to orbits and sinuses. A cervical biopsy of the enlarged lymph nodes was taken and a histopathological diagnosis of Burkitt lymphoma was made. Genetic analysis showed a *GNB1 *mutation, and the patient was diagnosed with Kabuki syndrome by a pediatrician, based on characteristic dysmorphic features. Treatment with steroids and chemotherapy was initiated.

## Introduction

Kabuki syndrome (KS) is a congenital anomaly-mental retardation syndrome that was first described by two groups in Japan [1,2]. They described a group of patients who had two characteristic facial features, skeletal anomalies, dermatoglyphic abnormalities, short stature, and mental retardation. The dysmorphic features are typically described as eversion of the lower lateral eyelid, arched eyebrows, sparse or with an interrupted course, lateral extension of the palpebral fissure, a depressed nasal tip, and prominent ears [2].

The prevalence of KS in the Japanese population has been estimated to be 1/32,000 [3]. Burkitt lymphoma (BL) is a rare and highly aggressive subtype of non-Hodgkin lymphoma (NHL), which constitutes a varied group of hematologic malignancies derived from B cell progenitors, T cell progenitors, mature B cells, mature T cells, or (rarely) natural killer cells. There are three types of BL depending on causes or regions. Endemic BL is associated with Epstein-Barr virus (EBV) in over 95% of the cases and represents the most common childhood cancer in geographical areas where malaria is endemic [4]. Sporadic BL represents 30-40% of childhood NHL in Europe and North America and is rarely associated with EBV infection. Immunodeficiency-related BL commonly mainly affects HIV/AIDS patients [4]. 

Lymphomas originating from the central nervous system (CNS) are termed primary CNS lymphomas (PCNSL), which are relatively rare, especially in immunocompetent people. Annually, the case number of PCNSL in the United States is seven per 1,000,000 people [5]. Acute sudden blindness is a rare presenting symptom of PCNSL or NHL in general [6]. There is also limited data on the association between lymphoma and KS. According to the best of our knowledge, there have been no reports of PCNSL in a KS patient.

## Case presentation

A four-year-old Saudi male born to first-degree consanguineous parents presented to the emergency department in our hospital in Jeddah, Saudi Arabia, with altered consciousness and a gradual decrease in vision for 15 days noticed by the mother. He was referred to the ophthalmology clinic for his ocular complaints. He was a known case of central hypothyroidism, developmental abnormality, and speech delay diagnosed in the first year of life. The patient was in his usual state of health until 20 days ago, when the mother noticed high body temperature, and upon visiting their primary health care it was documented as 38^o^C. The fever improved when given antipyretics. It was associated with upper respiratory tract symptoms and vomiting for which He was prescribed antibiotics at a local primary healthcare center and the symptoms improved.

Two days later, the mother noticed left eye deviation followed by orbital and facial swelling associated with bilateral eye proptosis and progressive decrease in vision, eventually becoming blind one week later. There was lymph node enlargement and pain in the neck associated with decreased oral intake and weight loss of 3 kg in the past two weeks. He had a positive history of an on-and-off headache, as the patient was observed to be crying and holding his head repeatedly by his parents, especially in the occipital area, for one month. Other systemic reviews were unremarkable. A review of past medical history was significant for one previous hospital admission at the age of four months due to viral respiratory illness. Vaccinations were up to age.

**Figure 1:**
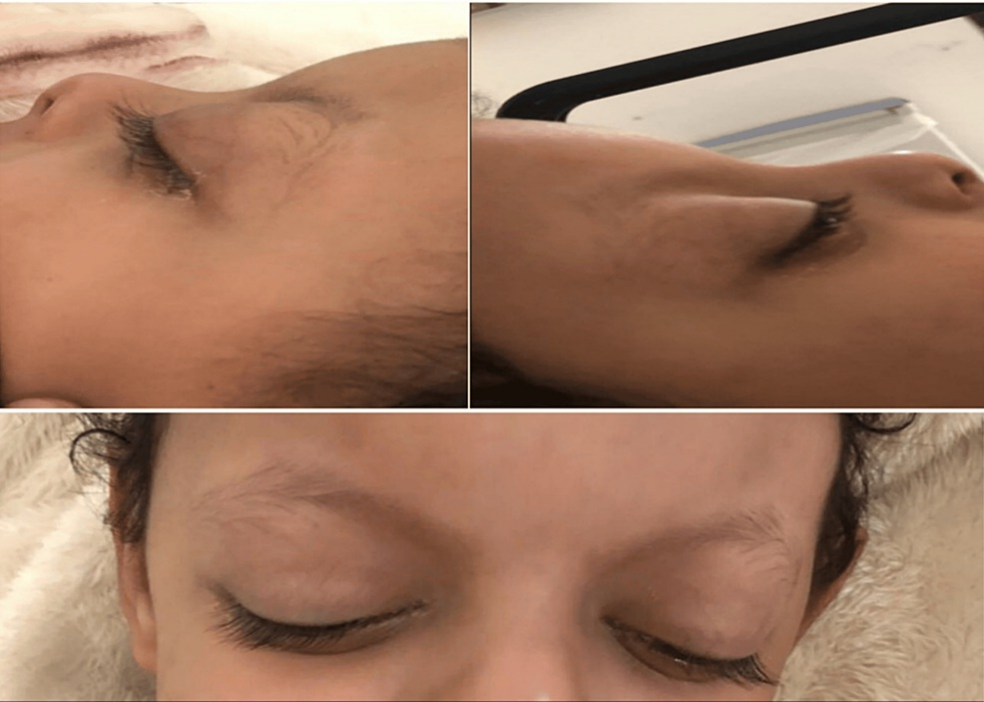
Bilateral proptosis, prominent eyelashes, arched, sparse, eyebrows, and a broad nose with a depressed tip. Permission to publish images was taken from the patient’s guardians.

On examination, the patient was conscious but not oriented. He showed a decrease in activity and altered mental status. He had dysmorphic features including prominent eyelashes, eversion of the lower lateral eyelid, arched, sparse, eyebrows, and a broad nose with a depressed tip. Four lymph nodes were palpable at the cervical-2 level. The abdomen was soft and lax, and there were no palpable masses or organomegaly. Ophthalmological examination showed no light perception vision. Both pupils were nonreactive and mid-dilated. There was bilateral proptosis of the eyes (Figure 1). There was limited extraocular muscle (EOM) movement, the eyes were fixed straight. Lids and lacrimal glands were within normal limits, the conjunctiva was quiet, the cornea was clear, no iris abnormality was seen, the anterior chamber was deep and quiet, the lens was clear, and fundus exam by indirect ophthalmoscope showed bilateral grade 3/4 papilledema, healthy macula with good reflex, normal retinal vasculature, and periphery.

**Figure 2:**
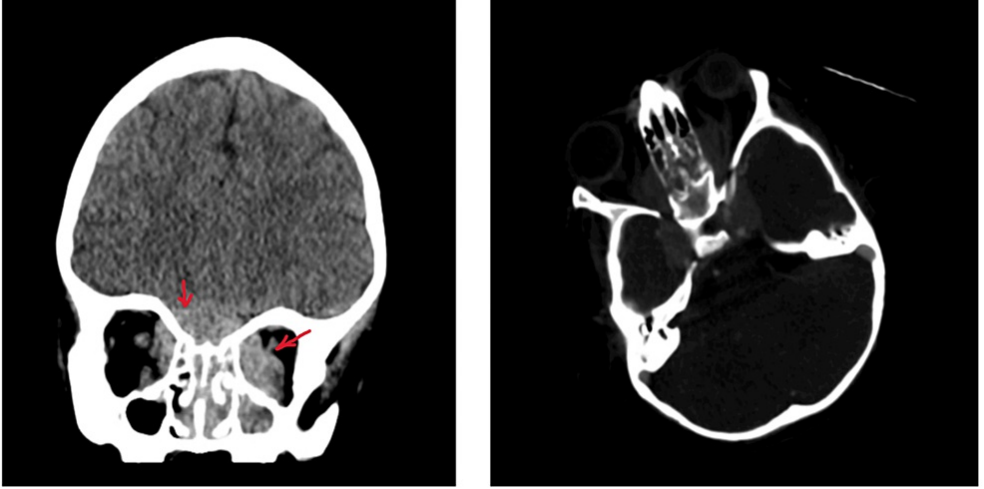
CT Brain showed a large enhancing mass in the base of the skull measuring 6.1 x 4.1 cm involving both anteromedial temporal and the inferior frontal lobes, cavernous sinus bilaterally, nasal sinuses and extending to the orbits bilaterally with mass effect on the optic nerve.

The patient was admitted and a CT brain was ordered, and results revealed a mass in the base of the skull extending to the orbits and sinuses (Figure 2). A biopsy of the enlarged cervical lymph node was taken and a histopathological diagnosis of BL was made. Flow cytometric analysis of neck lymph nodes showed around 72% monoclonal B-cells and 25% mature T-cells with no aberrant loss. A brain and orbit MRI was requested for detection of the origin and invasion of the tumor showed a large skull base mass involving the Sella turcica and pituitary gland with mass effect on the bilateral optic nerve. A bone marrow aspirate showed more than 25% involvement. Cerebrospinal fluid analysis (CSF) was normal.

On immunohistochemistry, tumor cells were positive for CD20, CD10, and Bcl-6. They were negative for Bcl-2, MUM-1, TDT, and CD34. T-cells were positive for CD3 and CD5. Ki-67 was positive in 99% of tumor cells. A thoracic and abdominal CT was done and showed no metastasis or organomegaly. Whole exome sequencing was performed by a medical geneticist for evaluation of dysmorphic features and suspicious speech delay which showed a GNB1 mutation, with underdetermined significance. Clinically, the patient was assumed to have KS by a pediatrician and clinical geneticist.

The patient was diagnosed with BL, Group C > 25% bone marrow, and CNS involvement on MRI, CSF negative. Treatment with chemoimmunotherapy using cyclophosphamide, vincristine, prednisone, doxorubicin, and rituximab was started on February 10, 2020, and finished on August 16, 2020, a total of six months. After one week of initiation of chemotherapy, the patient displayed mild improvement of proptosis in both eyes. The optic disc remained edematous in the left eye as grade 3/4, and there was no light perception during examination by direct ophthalmoscope. However, the right eye showed better results with a decrease in optic disc edema to grade 2/4, and the patient was able to perceive light. The patient was still unable to move his eyes. These clinical findings correlate with follow-up imaging. The brain MRI showed a tumor originating from the pituitary gland and extending toward the optic nerves and sinuses. However, in comparison to the previous image, there was a significant decrease in the size of the mass to around 1x1.5 cm and a decrease in the bilateral extraconal orbital medial soft tissue components with a reduction of the mass effect on the bilateral optic nerves.

Patient was followed up by a multidisciplinary team including Ophthalmology, Oncology, Endocrinology, and Pediatrics. From an Ophthalmology perspective, the vision didn't improve as no light perception was found on multiple follow-ups, the last one being in March 2024 and the patient was diagnosed with cortical blindness. 

## Discussion

This report described a patient with KS based on characteristic dysmorphic features, presenting with primary CNS BL originating from the base of the skull and extending to the orbits and sinuses. The tumor compressed the optic nerves causing bilateral sudden loss of vision and significant proptosis without systemic involvement. EOM limitation, proptosis, and mydriasis indicate multiple cranial nerve palsy due to mass effect and possible cavernous sinus syndrome. Isolated ocular manifestations without systemic involvement point to a significantly fast-growing, locally aggressive tumor. A previous report with ocular manifestations documented concomitant systemic involvement [7].

In addition, the sudden, simultaneous, and bilateral loss of vision in our case has rarely been reported in the literature. In most other cases, vision loss is unilateral or consequential. The origin of BL from the CNS is rare, especially in non-HIV patients [8]. The patient lives in a non-endemic region of Saudi Arabia for malaria, which has been linked with mature B cell cancers by eliciting protracted AID expression in germinal center B cells [9], leading to endemic BL. This may suggest that the patient had sporadic BL; however, the patient had a history of admission due to viral illness and a positive EBV-IgG result, thus endemic BL remains a likely possibility.

Several previous reports have associated KS with various types of cancers, suggesting a cancer predisposition. These reports include associations with hepatoblastoma, leukemia, neuroblastoma, synovial sarcoma, giant cell fibroblastoma, pilomatricoma, Wilms tumor, and desmoid fibromatosis [10-12]. Two previous reports have associated KS with BL originating from the abdomen and the rhinopharyngeal area [13,14]. However, this is the first case to report PCNSL in a KS patient to the best of our knowledge. It has been reported that individuals with KS have impaired immune systems [15], making these individuals more liable to recurrent infections and certain malignancies. Nevertheless, cancer remains a non-key feature of KS due to the paucity of reports in the literature.

## Conclusions

In this report, we presented a case of BL infiltrating the base of the skull including the pituitary gland causing sudden total blindness in a child with KS. We suggest more extensive studies be done to understand the biological basis of cancer predilection in KS patients and whether cancer screening protocols and ophthalmological baseline examination should be considered for them. Furthermore, KS is an important differential that should be included in the workup of patients with sudden vision loss.
